# Numerical Study of Metachronal Wave-Modulated Locomotion in Magnetic Cilia Carpets

**DOI:** 10.1002/aisy.202300212

**Published:** 2023-07-20

**Authors:** Hao Jiang, Hongri Gu, Bradley J. Nelson, Teng Zhang

**Affiliations:** Department of Mechanical and Aerospace Engineering Syracuse University, Syracuse, NY 13244, USA; BioInspired Syracuse Syracuse, University Syracuse, NY 13244, USA; Department of Physics, University of Konstanz, 78464 Konstanz, Germany; Institute of Robotics and Intelligent Systems, ETH Zurich, 8092 Zurich, Switzerland; Department of Mechanical and Aerospace Engineering Syracuse University Syracuse, NY 13244, USA; BioInspired Syracuse Syracuse University Syracuse, NY 13244, USA

**Keywords:** lattice models, locomotion, magnetic cilia carpets, metachronal waves, modeling and simulations

## Abstract

Metachronal motions are ubiquitous in terrestrial and aquatic organisms and have attracted substantial attention in engineering for their potential applications. Hard-magnetic soft materials are shown to provide new opportunities for metachronal wave-modulated robotic locomotion by multi-agent active morphing in response to external magnetic fields. However, the design and optimization of such magnetic soft robots can be complex, and the fabrication and magnetization processes are often delicate and time-consuming. Herein, a computational model is developed that integrates granular models into a magnetic–lattice model, both of which are implemented in the highly efficient parallel computing platform large-scale atomic/molecular massively parallel simulator (LAMMPS). The simulations accurately reproduce the deformation of single cilium, the metachronal wave motion of multiple cilia, and the crawling and rolling locomotion of magnetic cilia soft robots. Furthermore, the simulations provide insight into the spatial and temporal variation of friction forces and trajectories of cilia tips. The results contribute to the understanding of metachronal wave-modulated locomotion and potential applications in the field of soft robotics and biomimetic engineering. The developed model also provides a versatile computational framework for simulating the movement of magnetic soft robots in realistic environments and has the potential to guide the design, optimization, and customization of these systems.

## 1. Introduction

Metachronal motion is a coordinated, undulatory movement of multiple mechanically active segments found in many terrestrial and aquatic organisms, such as the moving legs of arthropods and the beating tentacles of Ctenophora.^[1-3]^ The rhythmic patterns allow the active segments to perform various collective functions that cannot be achieved by single individuals. For example, metachronal waves on cilia arrays can promote fluid pumping at the microscopic scale for efficient swimming of small organisms^[4]^ and convective transport of nutrients in reef corals.^[5]^ In another case, the same wavy pattern is used by millipedes to generate a strong thrust force in the direction of motion for burrowing, climbing, or walking.^[6]^ There is a growing interest in realizing metachronal patterns in artificial systems, despite the engineering challenges in complexity, fabrication, and system integration. For example, micropumps using artificial cilia with metachronal waves^[7-10]^ have been shown to be more efficient and effective at microfluidic pumping compared to identically in-phase beating ciliary micropumps. These systems have also been used to transport fluid,^[11-15]^ to manipulate microobjects^[16,17]^ and to create more resilient soft robots.^[18-25]^ Bio-inspired artificial systems have greatly enhanced our understanding of natural phenomena and offered valuable insights for a wide range of applications.

Among the many types of artificial metachronal realizations, hard-magnetic soft (HMS) actuators exhibit unique advantages and become the most popular artificial systems with metachronal waves. HMS actuators^[26-33]^ can be driven by an external magnetic field, which avoids complicated wiring to power and control of individual actuators (e.g., in electrical, pneumatic, and hydraulic actuator systems). In addition, the phase difference in the metachronal wave can be encoded as the magnetization of the individual HMS actuators. Their simple structure, easy fabrication, low cost, and reprogrammable feature enable HMS robots to be used in a wide range of applications,^[34-41]^ particularly for minimally invasive medical interventions and on-chip micromanipulations. However, current optimizations are based on simple analytical models that cannot fully capture the complex interactions between the robot and its environments. Millimeter- to centimeter-sized medical robots need to navigate in highly complex terrains (e.g., the gastrointestinal tract) with unpredictable disturbances (e.g., bowel movement). In on-chip micromanipulations, the HMS actuator arrays have both direct surface contact and indirect hydrodynamic interactions with the targeted object, where surface properties and boundary conditions are often unknown. Therefore, the optimization and customization of those HMS robots rely heavily on repetitive experimental trials and errors, which is very inefficient and time-consuming. To advance these applications, numerical simulations that can capture the complex interactions between the HMS robots and the environments are urgently needed. As in many robotic systems, reliable numerical simulations can accelerate the design and optimization of HMS robots, promoting their implementation in real-world applications.

One major challenge in modeling and simulating soft robots with HMS materials is their nonlinear material behavior and the coupling between mechanical deformation and magnetic actuation. Considerable efforts have been made to develop constitutive relationships for HMS materials to account for magnetoelastic effects, such as magnetically induced forces and deformations, mechanically induced changes in magnetization, and field-induced changes in the effective modulus.^[42-48]^ For example, microstructure-based models^[45,47]^ have been proposed to incorporate shapes and distributions of the magnetic particles or fibers in the homogenization process of deriving the effective constitutive laws. In contrast, phenomenological continuum models^[46,48]^ are developed starting from free energy functions of mechanical deformation, magnetic fields, and their coupling. To balance accuracy and efficiency, Zhao et al.^[46]^ derived a simplified model for HMS material when the material is premagnetized and subject to a relatively weak external magnetic field, which couples the mechanical stress and magnetic field without explicitly including the magnetic equations in the solver. In addition, this model was implemented in ABAQUS through the user-defined element (UEL) subroutine^[46]^ and the authors showed that it can accurately capture various nonlinear shape morphing of structured HMS materials. Following the same framework, Ye et al. developed a magnetic–lattice model (Magttice model),^[49]^ which utilized a finite-element scheme to compute lattice spring stiffness and incorporated the external magnetic field with precomputed nodal forces. The model is further implemented using the lattice Boltzmann method^[50]^ to simulate fluid–structure interactions, such as a swimming magnetic soft robot. Reduced models of slender hard-magnetic structures, such as shells and plates^[51-53]^ as well as rods,^[54-59]^ have been developed to further reduce the computational cost. They are very efficient and thus promising for real-time simulations and inverse design of robots.^[57,59]^

Another challenge is the nonlinear interactions at the interface between the soft robots and their environments, such as friction and adhesion. In many soft robotic systems,^[37,60-68]^ friction and adhesion play a critical role in locomotion performances, especially for those robots that can climb walls, hang from ceilings, and run on granular surfaces. At the moment, it is prohibitively challenging to experimentally access the dynamic contact forces between individual contacting points and the substrate with varying levels of friction and adhesion during locomotion. Therefore, many simulation methods have been developed to simulate interfacial interactions. For frictional forces, Coulomb friction model^[69,70]^ has been widely used due to its simplicity. It requires users to fit parameters by qualitatively matching the predicted gaits with experimental observations. Recently, Huang et al.^[59]^ applied the incremental contact potential method and discrete rod model to simulate the rolling and crawling of magnetic cilia carpet. Meanwhile, the discrete element method has been used to simulate walking and running of soft robots on granular substrates.^[71-73]^ The interplay between friction and adhesion is complicated to simulate even for relatively simple geometries,^[74]^ leaving it largely unexplored at the robotic system level.

**Figure 1.**
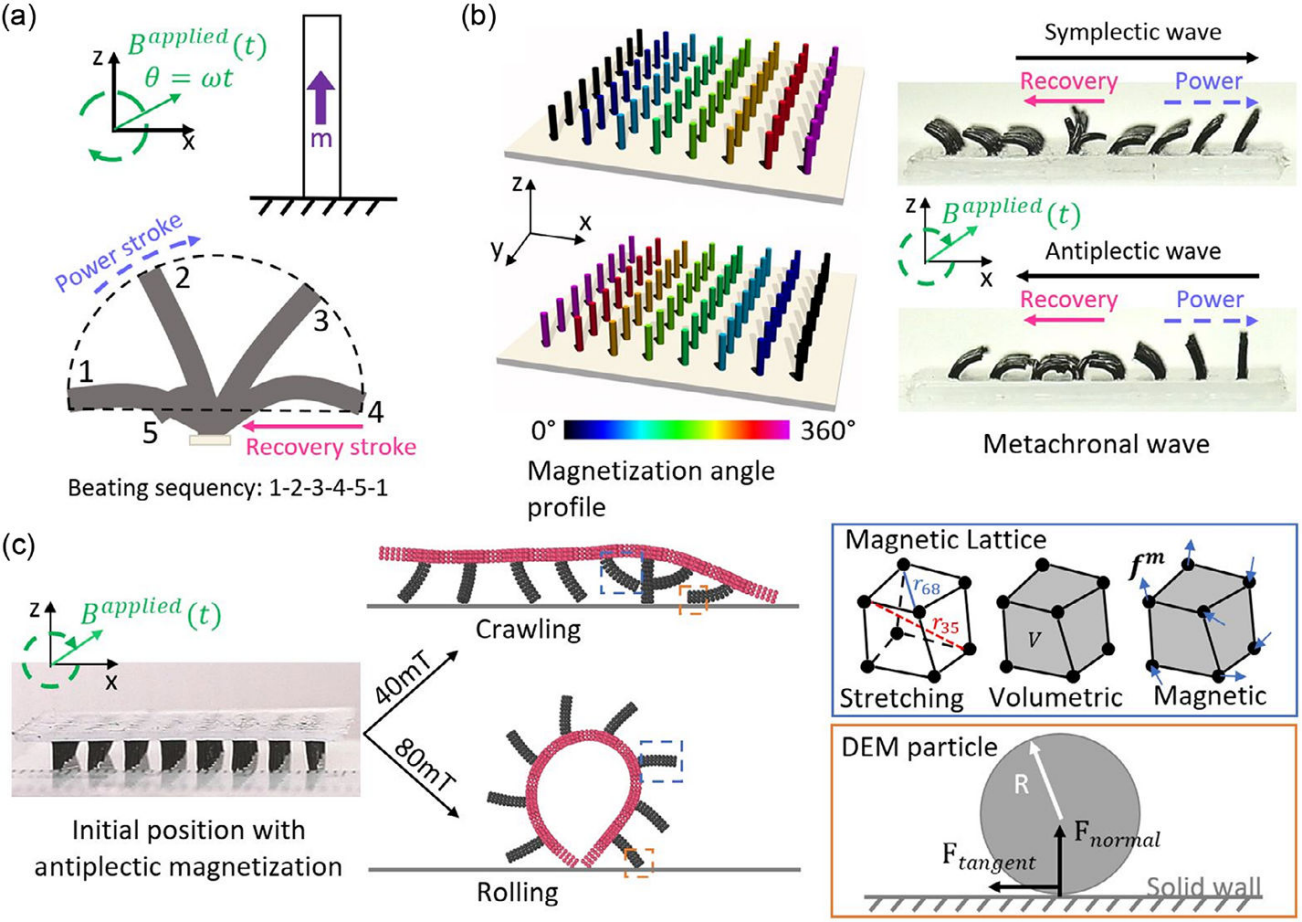
Modeling soft robotic locomotion (e.g., crawling and rolling) in magnetic cilia carpets with metachronal waves. a) Beating kinematics of a single cilium. b) Magnetization profiles for different metachronal waves. c) Simulation setup and representative snapshots of crawling and rolling locomotion where magnetic responsive materials and interface contact are described by the magnetic–lattice model (Magttice model) and the discrete element method model, respectively.

Despite this progress in modeling soft robots and HMS materials, there is still a lack of efficient and accurate computational platforms for modeling the terrestrial locomotion of magnetic soft robots. This is due to the complex coupling between the interfacial friction and the nonlinear deformation of magnetically responsive materials. To address these challenges, we propose a model that integrates the particle contact model of granular materials^[75,76]^ into the Magttice model.^[49]^ Both the Magttice model and the particle contact model have been implemented in the open-source molecular dynamics package large-scale atomic/molecular massively parallel simulator (LAMMPS),^[77]^ which is highly efficient for the parallel computing of large-scale simulations. In addition to the simple implementation, the high parallel efficiency of LAMMPS is maintained. Using the new method, we first investigate the influence of magnetization profile on single-artificial-cilium deformation under a rotating external magnetic field (Figure 1a). Then two types of metachronal waves, namely simplectic and antiplectic, are simulated by altering the magnetization profile on the artificial cilia array (Figure 1b). At last, the model is used to simulate metachronal wave-modulated locomotion of inverted magnetic cilia carpets, including both crawling and rolling (Figure 1c) motions. The simulated crawling speeds based on different metachronal wave vectors match well with the experimental measurements. Our simulations can further provide detailed spatial and temporal distribution of friction forces and gaits of each robot leg, which points out future directions to improve the robot crawling speed. The combination of the granular contact model and the Magttice model, along with previously established fluid–structure interaction coupling capability, provides a convenient and versatile computational framework for simulating magnetic soft robotic applications with more realistic environmental interactions. This can provide valuable insights into the design and control of magnetic soft robots and enable their practical use in a wide range of applications.

## 2. Computational Models

### 2.1. Magttice Model

Here, we give a brief review of the Magttice model and refer the readers to the literature for detailed derivations.^[49]^ The Magttice model numerically solves the deformation of structures made by a type of hard-magnetic materials, whose strain energy density is given by ^[46]^

(1) $$U=\frac{G}{2}\left(I_{1}-3\right)+{\frac{K}{2}(\text{ln}J)}^{2}-\text{Gln}J-\frac{1}{\mu_{0}}F{\tilde{B}}^{r}\cdot B^{\text{applied}}$$

where G is the shear modulus, K is the Bulk modulus, $I_{1}$ is the first invariant of the right Cauchy–Green deformation tensor, J is the determinant of the deformation gradient tensor F, $\mu_{0}$ is the vacuum permeability, and ${\tilde{B}}^{r}$ and $B^{\text{applied}}$ denote the residual and applied magnetic flux densities, respectively. In the simulations, a continuum solid will be first discretized into 8-node hexahedral elements. With the aid of finite-element shape functions, $N^{a}$, a=1,2,…,8, the mechanical strain energy density within one element can be rewritten as a summation of stretching of the lattice that connects two vertexes and volumetric change of the element^[49]^

(2) $$U_{\left(I_{1}\right)}=\frac{1}{2}V_{0}^{-1}\sum_{b=2,b>a}^{8}\sum_{a=1}^{7}k_{ab}r_{ab}^{2}-G$$

(3) $$U_{J}={\frac{1}{2}K(\text{ln}(V∕V_{0}))}^{2}-G\text{ln}(V∕V_{0})$$

where $r_{ab}=x^{a}-x^{b}$, and $k_{ab}=-\int G\frac{\partial N^{a}}{\partial X_{j}}\frac{\partial N^{b}}{\partial X_{j}}$, j=1,2,3, a=1,2,…,7, b=2,3,…,8, is the lattice stiffness that can be precomputed before the simulations; V and $V_{0}$ represent the volumes of the lattice at the deformed and reference configurations, respectively. Hexahedral elements are chosen to describe the near incompressibility of elastomers, which is achieved by setting a large bulk modulus, such as K=20 G. Similarly, the magnetic energy density can also be precomputed as

(4) $$U_{\text{magnetic}}=V_{0}^{-1}\left(-f_{m}\right){}_{i}^{a}x_{i}^{a}$$

where ${\left(f_{m}\right)}_{i}^{a}=\frac{1}{\mu_{0}}{\tilde{B}}_{j}^{r}B_{i}^{\text{applied}}\int \frac{\partial N^{a}}{\partial X_{j}}\;dV_{0}$, i, j=1,2,3, a=1,2,…,8, can be seen as general nodal forces associated with the magnetic actuation (Figure 1c). For a nonuniform external magnetic field, a body force associated with the gradient of the external magnetic field is also needed to extend Equation (4), making the computation more complicated. One possible direction is to adopt a tetrahedron element to create a constant gradient in the element, which can leverage recent progresses of simulations of hard magnetic materials under a constant gradient magnetic field.^[55,58]^

### 2.2. Frictional Contact Model

The frictional contact is realized by integrating the contact models from the granular package of LAMMPS into the Magttice model. This coupling is achieved by defining the lattice nodes as granular particles which enables a variety of built-in contact models associated with the granular pair style (Figure 1c). The contact force between two bodies or one body and a wall can be effectively modeled through the defined forces by the specified contact models,^[77]^ e.g., “Hooke”, “Hertz”, “DMT”, and “JKR” for normal contact force; “Linear_nohistory”, “Linear_history”, and “Mindlin” for tangential contact force. For current simulations of ciliated soft magnetic robot locomotion, the adhesive DMT normal contact model and Mindlin tangential contact model are selected and shown to capture various locomotion of magnetic cilia carpets in the experiment.^[20]^ Other combinations of the contact models may also achieve the same performances. The comparison of different friction models requires more systematic studies and is a very important future research direction. The DMT model corresponds to the Derjaguin–Muller–Toporov cohesive model,^[75,76,78]^ the elastic component of the normal force between two contact bodies i and j can be written as

(5) $$F_{\text{ne}}=\left(\frac{4}{3}E_{\text{eff}}R_{\text{eff}}^{1∕2}\delta^{3∕2}-4\pi \gamma R_{\text{eff}}\right)n$$

where $E_{\text{eff}}={\left(\frac{1-\nu_{i}^{2}}{E_{i}}+\frac{1-\nu_{j}^{2}}{E_{j}}\right)}^{{}^{{}^{{}^{{}^{-1}}}}}$ is the effective Young’s modulus calculated using modulus ($E_{i}$, $E_{j}$) and Poisson ratios ($\nu_{i}$, $\nu_{j}$) of particle i and j, δ is the overlap of the contact, γ is the surface energy density, and $R_{\text{eff}}=\frac{R_{i}R_{j}}{R_{i}+R_{j}}$ is the effective radius. Note that the body–wall contact is approximated by treating the wall as a particle with an infinite radius. In such case, $R_{\text{eff}}$ is just equal to the radius of the particle on the body. In the simulations, every node in the lattice is assigned a constant radius value of three-quarters of the mesh size. In addition, the normal force is augmented by a damping term

(6) $$F_{n,\text{damp}}=-\eta_{n0}m_{\text{eff}}\nu_{n,\text{rel}}n$$

where $\eta_{n0}$ is the normal damping coefficient, $m_{\text{eff}}=\frac{m_{i}m_{j}}{m_{i}+m_{j}}$ is the effective mass, and $v_{n,\text{rel}}$ is the relative velocity between particle i and j along the normal direction.^[77]^ The total normal force is computed as the sum of the elastic and damping components

(7) $$F_{n}=F_{\text{ne}}+F_{n,\text{damp}}$$

The tangential force defined by the Mindlin contact model^[79]^ is given by

(8) $$F_{t}=-\text{min}(\mu_{t}F_{n0},\;|\;-k_{t}\alpha \xi +F_{t,\text{damp}}|)t$$

where $\mu_{t}$ is the friction coefficient, $F_{n0}=|F_{\text{ne}}+2F_{\text{pulloff}}|$ with $F_{\text{pulloff}}=4\pi \gamma R_{\text{eff}}$, $k_{t}$ is the shear stiffness coefficient, $\alpha =\sqrt{\delta }R_{\text{eff}}$ is the radius of contact region, $\xi =\int_{t_{0}}^{t}v_{t,\text{rel}}(\tau )d\tau$ is the accumulated tangential displacement during the contact duration, $F_{t,\text{damp}}=-\eta_{t0}m_{\text{eff}}v_{t,\text{rel}}$ is the tangential damping force with $\eta_{t0}$ as the tangential damping coefficient, and $v_{t,\text{rel}}$ is the relative velocity between particles i and j along the tangential direction. In the simulations, $\eta_{t0}$ is set equal to $\eta_{n0}$. The modified normal force $F_{n0}$ is to account for the effect of adhesion on the tangential forces.^[75,76]^

To couple the deformable lattice model and granular model in LAMMPS, the “special_bonds” command^[77]^ should be used to control the range of granular interactions between nodes in the body, such that interactions between neighboring nodes connected with bonds can be turned off while interactions between two surface nodes from different sections of the body are preserved. This configuration ensures that elastic deformation is determined only by the Magttice model and prevents penetration upon contact between different parts of a single body. It should be pointed out that other model combinations are also possible to achieve the same performances but have not been tested in the current work.

## 3. Simulation and Result

The magnetic soft robot studied in this work is in a form of a magnetic cilia carpet consisting of a nonmagnetic soft substrate and an array of magnetic cilia on top. Each of these cilia can move in different phases according to its own assigned magnetization angle. By coordinating their motion, i.e., creating a magnetization pattern along the direction of the cilia array, different metachronal waves can be generated.^[20]^ To investigate the mechanism of the soft robot locomotion, single-magnetic-cilium deformations and metachronal wave motions are first tested using our simulations to validate the model. Then, we simulated the soft robotic locomotion in inverted magnetic cilia carpets, including rolling and crawling.

### 3.1. Single-Cilium Study

**Figure 2.**
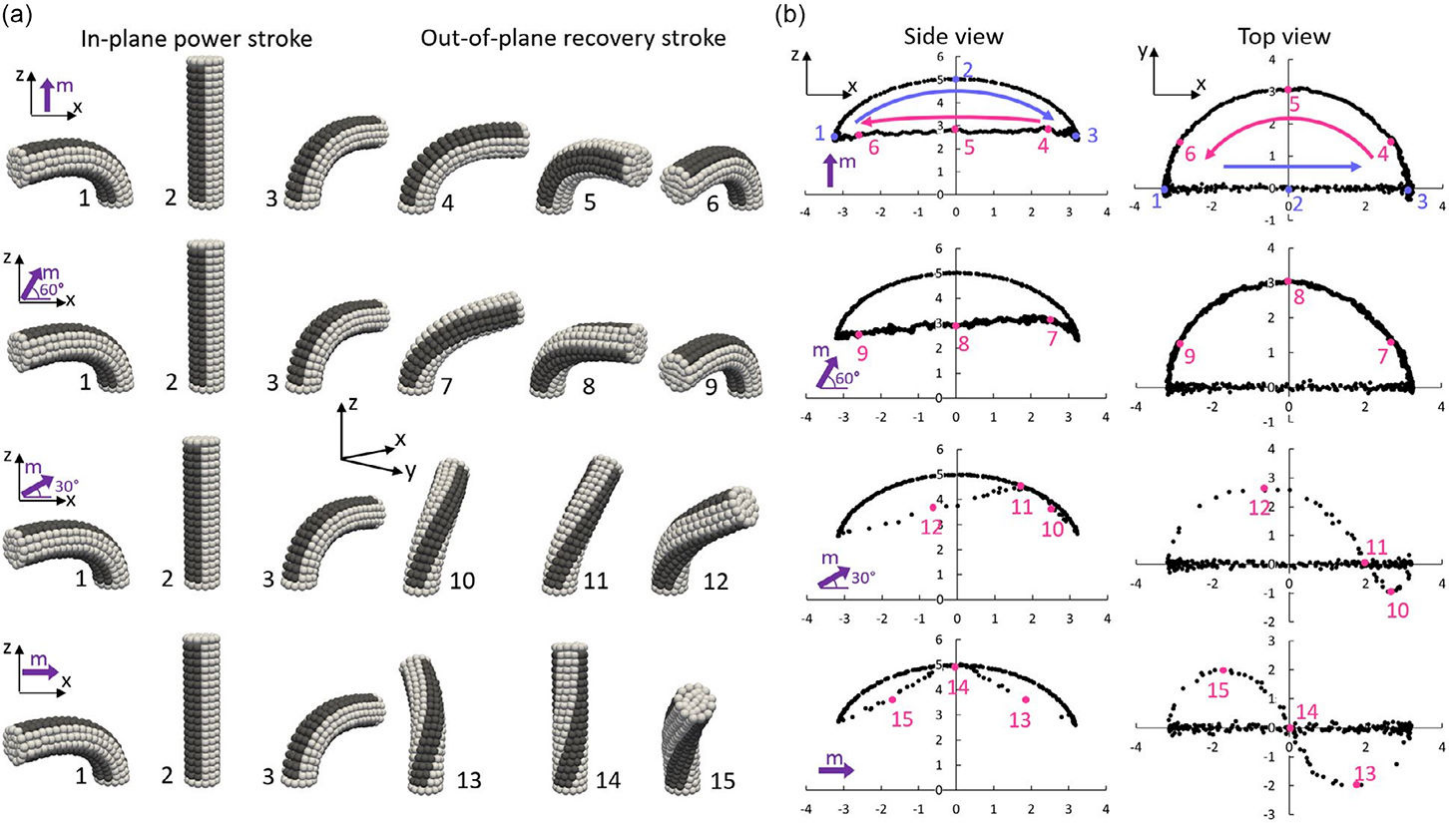
Single-cilium motion with different magnetization angles. a) Simulation snapshots of cilium deformation with 0°, 30°, 60°, and 90° magnetization angles, under 80 mT rotating magnetic field in the x−Z plane. b) The tip trajectories (under the side and top views) of the cilia motion with the corresponding magnetization angles from (a). Dark bands in (a) are only for visual guidance of the twisting and bending deformation.

In the simulation, the cilium is represented as a cylindrical pillar with dimensions of ø0.8 mm × L4 mm. This pillar is discretized into 400 elements and 567 nodes, and the bottom nodes are fixed in place. A harmonic wall boundary condition is applied at the lower boundary of the domain to prevent node penetration during the simulations. The cilium has mechanical and magnetic properties that are identical to those used in the experimental study,^[20]^ including a Young’s modulus of 185 kPa, a shear modulus of 61.6 kPa, a density of 2.39 g cm^−3^, and a residual magnetic flux density of 20 kA m^−1^. The cilium is actuated by a rotating magnetic field ($\omega =30^{{}_{{}^{\circ}}}\;s^{-1}$) in the x−z plane with a magnetic flux density of 80 mT. Four magnetization angles (0°, 30°, 60°, 90°) with respect to the x-axis in the x−z plane are tested to study the deformation and motion of single cilium, as shown in Figure 2. According to the simulation results, the motion of the cilium can be divided into two phases: the power stroke and the recovery stroke. During the power stroke, all four magnetization angles exhibit the same in-plane deformation and motion, with the direction of the residual magnetic flux density encoded in the cilium following the external magnetic field and bending within the x−z plane. However, when the cilium can no longer follow the rotating external magnetic field due to material and boundary constraints, it snaps out of the x−z plane, resulting in torsion and twisting in the cross section of the cylindrical cilium, as shown in Figure 2a. During the recovery stroke, the deformations and motions of the cilium depend on the magnetization angle. If the magnetization angle is along the cilium length, the force component in the z direction will maintain the degree of bending. However, if the cilium has a magnetization along its short axis, the twisting of the cilium causes oscillations of the force component in the z-direction, altering the degree of bending, as shown in the cilium tip trajectories plotted in Figure 2b. These changes in the cilium’s trajectory, from a “D”-shaped to an “8”-shaped path, are also observed in experiments with decreasing magnetization angles, and our simulation results show good agreement with experiments^[20]^ under the same test configurations (Figure S1, Supporting Information).

It is worth noting that, when the encoded cilium is under an external magnetic field that is perfectly in-plane (as in the simulations), the choice of which side of the x−z plane, the cilium will snap back to is random. This is because the two snap directions are symmetric and thus have an equal chance to happen. We observed both snapping directions in our simulations due to small random perturbations (Video S1, Supporting Information). The snapshots in the figure are all taken in a cycle where the cilium snaps back to the positive y side of the x−z plane for better visual comparison. It is possible to control which side the cilium snaps back to by slightly tilting the plane of the external field just before the snap occurs.

### 3.2. Metachronal Wave Study

**Figure 3.**
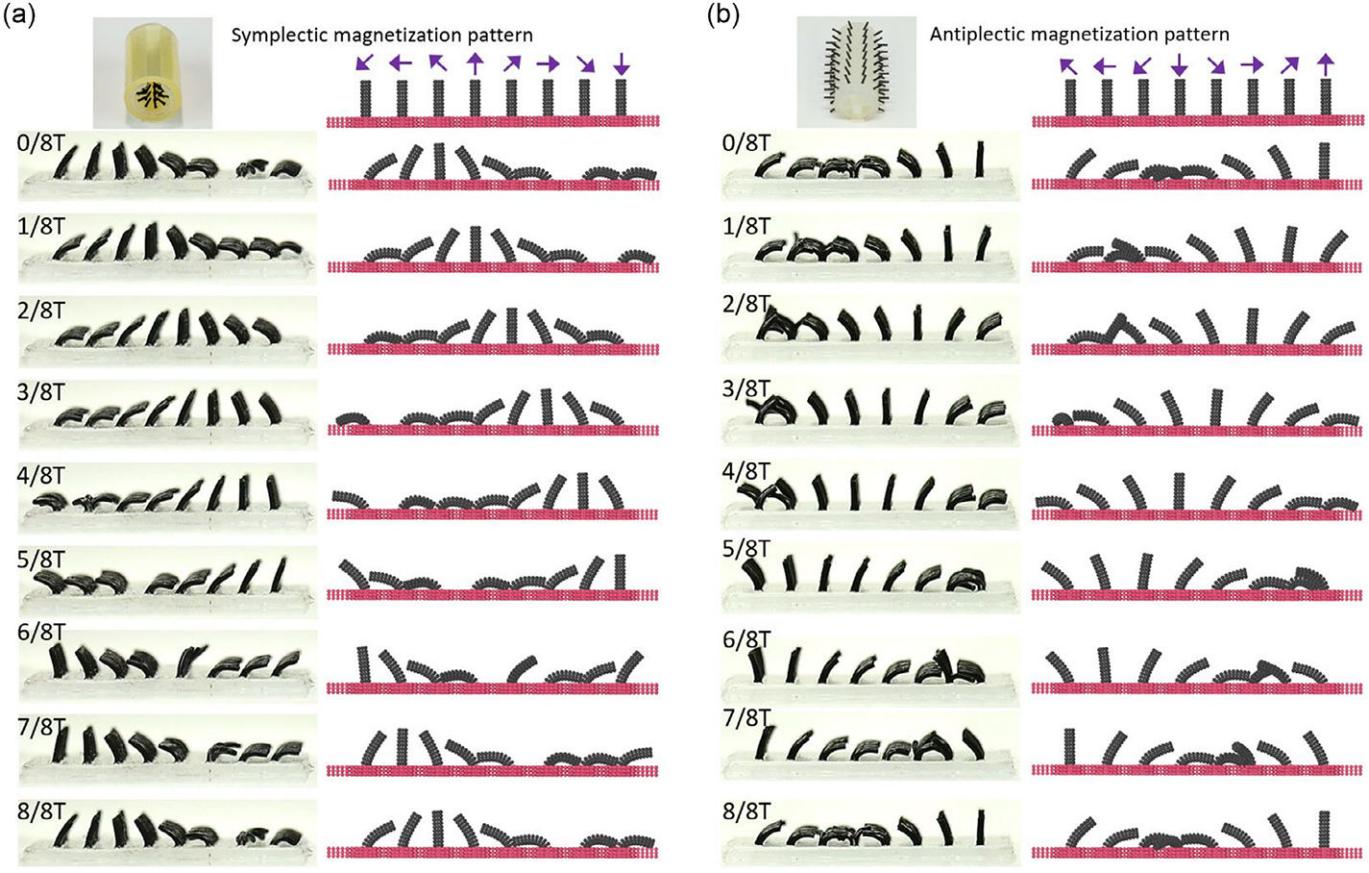
Comparison of simulation and experiential metachronal waves on magnetic cilia carpets under 80 mT rotating magnetic field. a) Symplectic wave. b) Antiplectic wave. Experimental images are adapted from ref. [20].

Depending on the relative direction of wave propagation to the power stroke, both symplectic and antiplectic metachronal waves can emerge on the cilia carpets. In a symplectic wave, the wave propagates in the same direction as the cilia’s beating (i.e., power stroke) direction, while an antiplectic wave propagates in the opposite direction (Video S2, Supporting Information). To create both metachronal waves in a cilia array under the same rotating external magnetic field, a specific magnetization pattern must be assigned to the cilia array. Figure 3 illustrates two magnetization patterns for generating symplectic and antiplectic wave motions in a cilia array, along with a comparison of the wave motion between experiments and simulations. The cilia array in this study consists of eight individual cilia (ø0.8 mm × L4mm) attached to a 36 mm long and 0.8 mm thick substrate, with a cilium-to-cilium distance of 4 mm. The discretization of the cilia is the same as the one in the single-cilium study. The mesh size of the substrate is about 0.2 mm, and the bottom nodes are fixed in space.

In the experiments,^[20]^ the cilia array is rolled and wrapped on the surface of a mold for the magnetization process. By controlling the curvature of the mold and the cilia facing inward or outward of the curved surface, different metachronal wavelengths and types can be encoded in the cilia array. Here, since the array is wrapped into a complete circle, the metachronal wavelength is equal to the perimeter of the circle (i.e., the length of the substrate). The resulting magnetization direction of each cilium is illustrated by the purple arrow in Figure 3. Snapshots for both experiments and simulations are taken at 1.5 s intervals over one period of a rotating magnetic field of 80 mT at 30° s^−1^ clockwise in the x−z plane. In each snapshot, the external magnetic field direction matches the magnetization direction of the cilium standing straight. This feature can be used to identify the wave’s traveling direction by finding the next straight cilium that appears on one side or the other of the current one. By sorting the sequence of straight cilia as time increments, we can see that the wave is propagating from left to right in Figure 3a and from right to left in Figure 3b. Since the power strokes in both cases are left to right, as the cilia follow the clockwise rotation of the external magnetic field above the substrate, this results in a symplectic metachronal wave in Figure 3a and an antiplectic metachronal wave in Figure 3b. Our numerical simulations agree well with the experimental measurements.^[20]^

### 3.3. Locomotion of Magnetic Cilia Carpets

**Figure 4.**
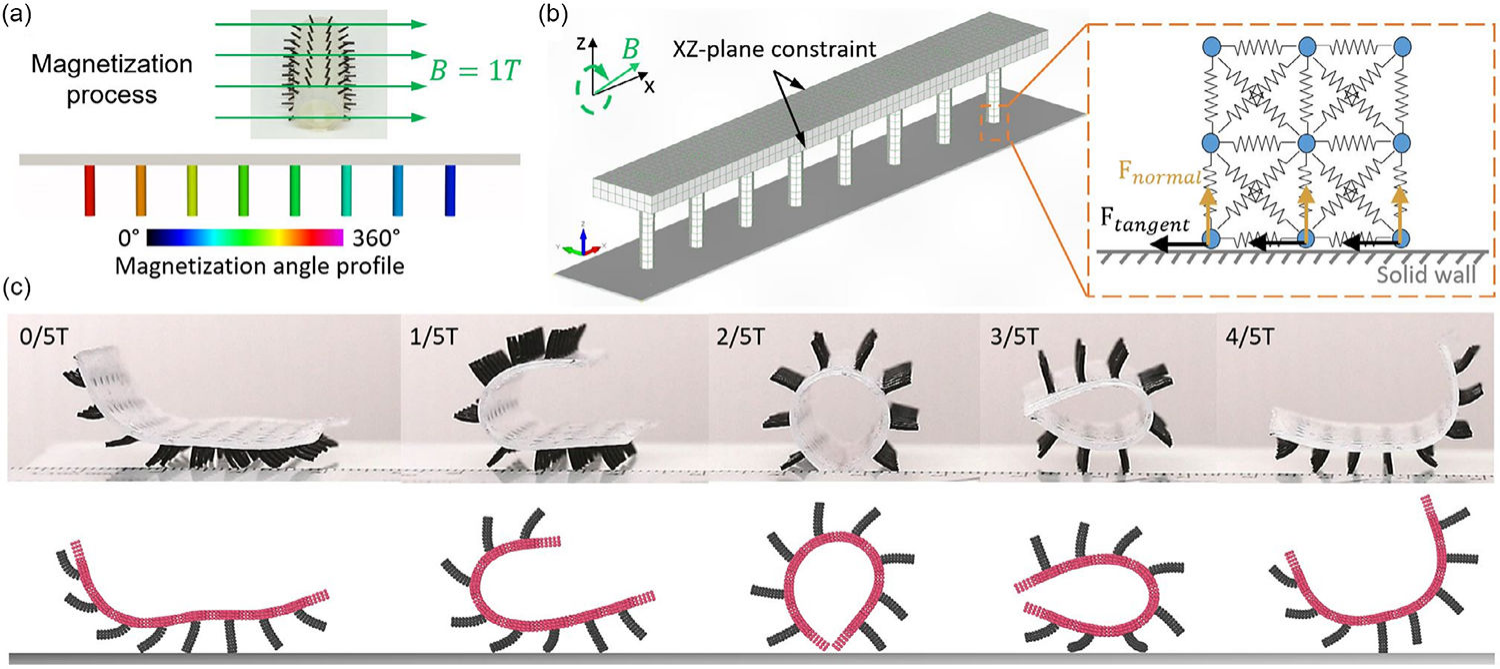
Simulations of the robot rolling. a) Magnetization process and magnetization angle profile. b) Simulation setup and boundary conditions. c) Posture comparison between experimental images and simulation snapshots within one cycle of external magnetic field rotation. Experimental images in (c) are adapted from ref. [20].

To this end, the single cilium, and the cilia array metachronal wave motions have been characterized by numerical studies with different magnetization profiles using the cilia on top of a flexible substrate. The nice agreements with experimental results prove the validity of our simulation model. The traveling metachronal wave generated by cilia motion resembles the leg moving pattern of a walking giant African millipede. The magnetization profile and applied external magnetic field can be used to control the locomotion gaits, which provide great potential for such robots on unstructured surfaces. The rolling and crawling of ciliated soft magnetic robots have been reported in the experiments, but there are no simulations to contribute to such unique robotic locomotion. Here, we apply our models to these complex locomotion and adopt the same mechanical and magnetic properties of the materials in Section 3.1 and 3.2. To calibrate the contact parameters, we run a number of simulations with different parameter sets to match the experimental measurements, such as the crawling speed at metachronal waves. The parameters we identified are shear stiffness coefficient $k_{t}=1.5\;\text{GPa}$, friction coefficient $\mu_{t}=0.45$, damping coefficient $\eta_{n0}=\eta_{t0}=0.01\;s^{-1}$, and surface energy density $\gamma =1.5\;{\text{J m}}^{-1}$, which are used for all the simulations in this section. For soft cilia carpet rolling, an antiplectic wave with a wavelength equal to the length of the substrate is encoded to the cilia carpet as shown in Figure 4a. This magnetization profile has the ability to roll the carpet into a tube with cilia facing outward under a uniform external magnetic field. In the rolling simulation, the same mesh used in the previous metachronal wave study is adopted to model a strip of the soft cilia carpet that is confined in 2D. The mesh is inverted in the z direction with the cilia pointing downward. The lattice nodes on the sidewalls of the substrate in the y-direction are constraints in the x−z plane while a solid wall is set at the bottom of the simulation domain enabling granular contact with the lattice nodes close to the wall, see Figure 4b. An external magnetic field of 80 mT points upward initially and rotates at 30° s^−1^ clockwise in the x−z plane as the simulation begins. The gravity force is applied as nodal forces on lattice nodes. The contact force can be decomposed into normal force and tangential force. For the DMT contact model used in this simulation, an adhesion term in the normal force can influences the magnitude of the tangential force as discussed in the previous section.

The snapshots in Figure 4c compare the gestures of the ciliated soft robots in experiment and simulation at constant time intervals from a rotation cycle. Initially, the cilia carpet is lifted at both ends as they attempt to align their magnetization direction with the upward-pointing magnetic field. As the magnetic field rotates clockwise, they follow the rotation. The cilia at the left ends are lifted further and the cilia at the right ends are dropped. As a result, the unmagnetized substrate is bent and folded from left to right. Once the external magnetic field rotates to the horizontal direction, the cilia carpet is rolled into a tube as the mold shape in the magnetization process in Figure 4a. The circular cross section allows the carpet to roll forward as the external magnetic field continues to rotate and the rolled carpet can unfold itself during the second half of the rotation cycle with the help of gravity (Video S3, Supporting Information). In the experiment, the rolling behavior occurs when the magnitude of the external magnetic field is larger than 60 mT. To make the soft cilia carpet roll, the applied magnetic field needs to generate sufficient force at the cilia to lift and bend the carpet for a circular cross shape. A similar threshold for the magnitude of the external magnetic field is also predicted by the model. Slight discrepancies in the trajectories of individual cilia can be observed in Figure 4c. One possible reason can be the simplification of the model, which only considers one row of cilia. In addition, simulation parameters may be further optimized to reduce the discrepancies.

**Figure 5.**
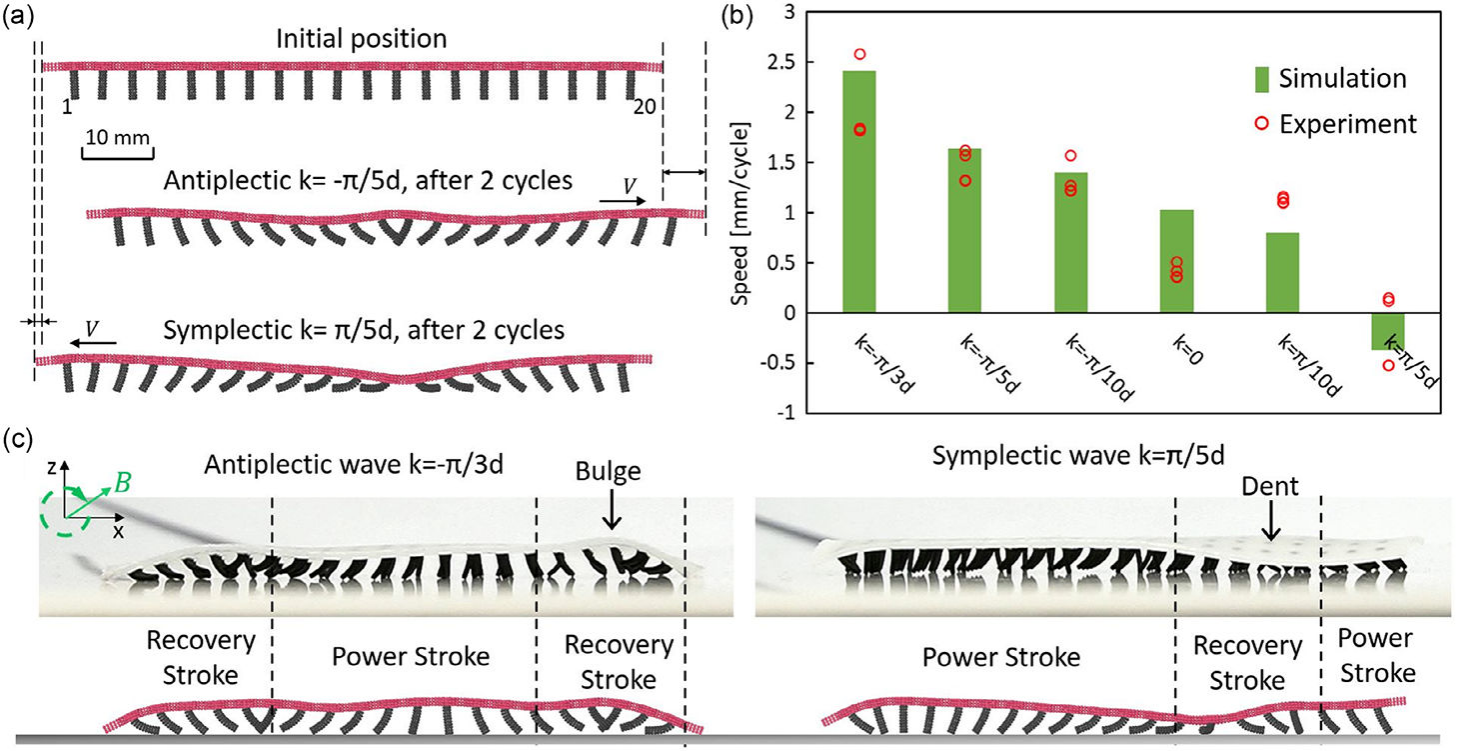
Impact of metachronal wave vectors on the crawling speed of soft robots. a) Comparison of crawling distance between antiplectic and symplectic wave encoded soft robots at the same wavelength, λ=10d. b) Crawling speeds of soft robots with different metachronal waves from simulations and experiments.^[20]^ c) Comparison of substrate deformation on antiplectic (λ=6d) and symplectic wave (λ=10d) soft robots between experiments^[20]^ and simulations.

The model is also used to investigate the influence of the metachronal wave vectors on the crawling speed of the ciliated soft robots. The cilia carpet strip used in the simulation consists of 20 individual cilia (ø0.8 mm × L4 mm) attached to a 124 mm long and 0.8 mm thick substrate. The cilium-to-cilium distance d is 4 mm. The mesh sizes and boundary conditions are consistent with the ones used in the rolling simulation. The magnetic field has a magnitude of 40 mT and rotates 30° s^−1^ clockwise in the x−z plane, which is adopted from the experimental settings. Six magnetization profiles are tested in the simulations with metachronal wave vector k ranging from −π∕3d to π∕5d. The magnetization angle of each cilium from left to right (with indices from 1 to 20) can be written as

(9) $$\theta =\theta_{0}+k\left(i-1\right)d,\;\;i=1,2,3,\ldots ,20$$

where $\theta_{0}$ is the magnetization angle of the most left cilium with an index equal to 1, k is the wave vector, i is the cilium index, and d is the cilium-to-cilium distance. Note that the negative wave vector results in antiplectic metachronal wave motion while the positive wave vector generates simplectic metachronal wave motion. For the case of k=0, all cilia motions are synchronized which acts as a control sample in this study. The granular wall contact boundary condition is the same as the one used in the rolling simulation. The crawling simulations of different metachronal wave vectors are compared with experiments in terms of the crawling speed per cycle as shown in Figure 5b. The predicted speeds and the experimental measurements are comparable and have the same trend as the wave vector increases. In general, soft robots with antiplectic waves exhibit higher crawling speeds than the ones with symplectic waves or with synchronized motion. Among six tested cases, the antiplectic wave vector of k=−π∕3d gives the fastest speed per cycle around 2.5 mm cycle^−1^ (Video S4, Supporting Information). As the wave vector increases, the speed decreases. With the same wavelength, λ=10d, antiplectic and symplectic wave-encoded soft robots can crawl in opposite directions, as shown in Figure 5a. One possible explanation is that the substrate dents during the recovery stroke under symplectic waves which enforces the contact with the frictional force opposite to the robot moving direction. On the contrary, antiplectic soft robot bugles during the recovery stroke and allows the cilia to move forward effectively, as shown in Figure 5c.

## 4. Discussion

**Figure 6.**
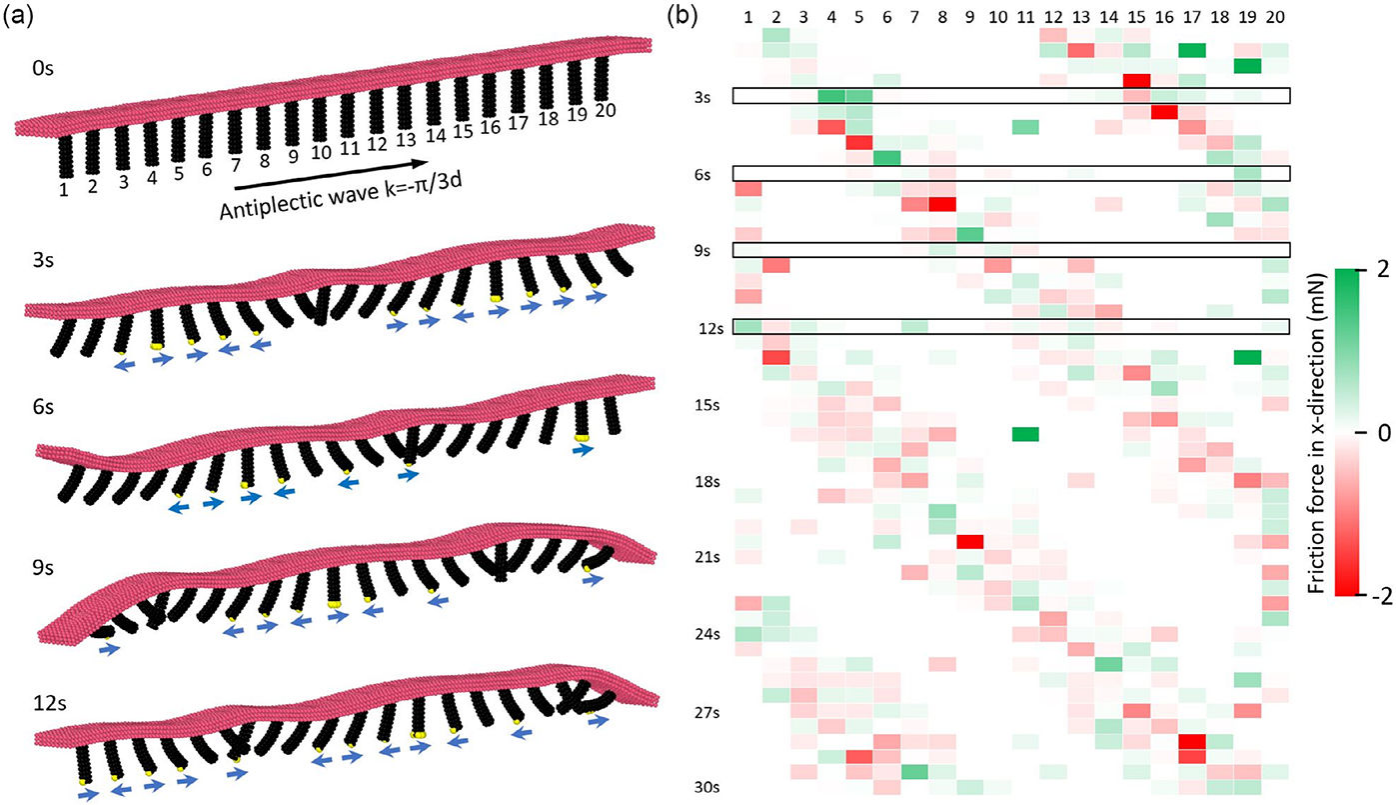
Friction forces during the soft robot crawling. a) Simulation snapshots of the soft robot crawling within one cycle of external magnetic field rotation, $\omega =30\;s^{-1}$. The cilia(legs) of the soft robot are magnetized to achieve an antiplectic metachronal wave with wavelength λ=6d. The simulation nodes with friction force exerted are colored in yellow and the blue arrows indicate the friction force directions. b) The x component of friction force on individual cilium(leg) over time. The cilium indices are mapped out in (a) and the boxed rows display the friction force distributions for each simulation snapshot’s moment presented in (a).

By integrating the granular contact model with the Magttice model in LAMMPS, our model successfully simulated the rolling and crawling locomotion of the magnetic-field-driven ciliated soft robot. In the previous examples, we have shown that the Magttice model is powerful to describe soft cilia deformation under magnetic actuation. Beyond that, the simulations can reveal detailed data that do not have access during the experiments. The granular contact model introduces friction between the cilia and the ground helping the robot to maintain balance and move forward. For robots that are designed to walk or crawl, the friction force is an important consideration in the design and operation. The robots rely on friction to maintain traction and stability as they propel themselves forward. Our model can be used to analyze the detailed friction force history of individual legs, as shown in Figure 6. The simulated robot in the presented example is magnetized with an antiplectic metachronal wave pattern of a wavelength equal to 6d. This means there are about 1.5 waves residing along the robot’s length. Figure 6a shows the detailed friction force history between individual legs with the substrate during the crawling motion with a period. The snapshots are taken every 3 s. In each snapshot, the nodes that experience contact forces are colored in yellow and the directions of the frictional forces are indicated by the blue arrow below the corresponding cilium. The direction of the frictional force is determined by the relative movement between the cilium and the ground. This movement has two contributions: one is the movement of the individual cilium caused by the magnetic actuation and the other is the drag caused by the moving of the entire robot. Therefore, even if the cilium is doing a power stroke, its relative motion to the ground may cause frictional force that prevents the robot from moving forward. At any transient moment, the number of cilia in contact with the ground has been influenced by the deformation of the individual cilium as well as the deformation of the substrate. The matrix in Figure 6b presents the × component of the friction force on each cilium in 2.5 cycles. The column represents the cilium index and the row represents the time. At any moment, there are two separate bunches of cilia that are in direct contact with the ground. A cilia contact wave pattern can be found in this sparse matrix as the contact cilia alternate from the left of the array to the right.

**Figure 7.**
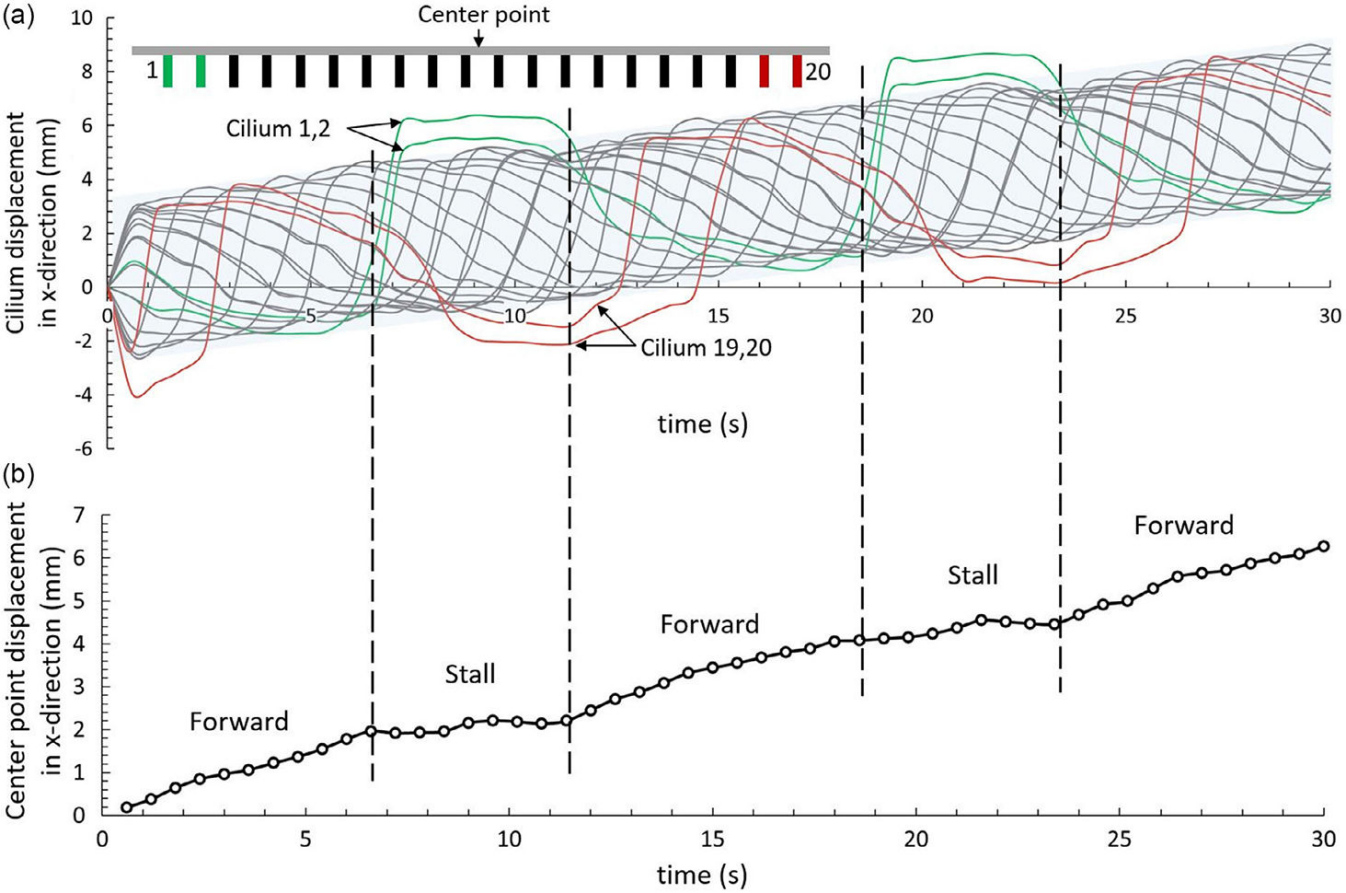
Crawling robot gaits and displacement. a) The trajectory of tips of each cilium(leg) under the side view, i.e., the displacements in the x-direction. The two front-end cilia are colored in red, while the two back-end cilia are colored in green. The displacements of the cilia in between are confined in a shadowed band with a span of less than 2 L, where L is the length of the cilium(leg). b) The displacement of the center point in the x-direction. The dashed lines separate the periods of forwarding and stalling.

In another example, the simulation results provide us with a better understanding of the walking gait with metachronal waves. The gait of the soft robot crawling can be studied by the trajectory of the free end of each cilium as shown in Figure 7. It is found that the displacement of each cilium in the x-direction is constrained in a band with a span about two times its length except the four cilia (denoted by green and red cilia in Figure 7a) at both free ends of the robot. The extra displacements for these four cilia come from the downward bending of the substrate. As the free ends of the substrate touch the ground, they continue to be pulled by the inward-pointing cilia causing large normal contact forces. It acts like a brake stopping the robot from moving. Figure 7b shows the displacement of the center point of the soft robot. The robot crawls forward in the first half of the period and stalls in the second half of the period. It would give a significant boost in terms of moving speed if the issue can be resolved, e.g., by increasing the stiffness of the substrate at two ends to prevent them from reaching the ground.

## 5. Conclusions

We propose an approach to simulate magnetic soft robots locomotion using the Magttice model for HMS material deformation and the granular model for frictional contact. Both models have been implemented in the highly efficient parallel computing platform LAMMPS. Integration is achieved by defining the lattice nodes as granular particles which enables a variety of built-in contact models associated with the granular pair style. The developed model accurately reproduces the deformation of single cilium, the metachronal wave motion of multiple cilia, and the crawling and rolling locomotion of magnetic cilia carpets. By encoding different metachronal wave vectors to the crawling soft robot, the simulations provided comparable moving speeds to the experimental measurements. Meanwhile, valuable information such as spatial and temporal variation of friction forces and trajectories can be obtained from the simulation for further analysis and optimization, which is difficult to do in the experiment. In addition, the current integration of the granular contact model to the Magttice model is compatible with its previously extended fluid–structure interaction coupling scheme,^[80]^ which provides a convenient and versatile computational framework for simulating soft magnetic robotic applications with more realistic environmental interactions. This can provide insights into the design and control of magnetic soft robots and enable their practical use in a wide range of applications. As future studies, our simulation framework can be further extended to describe HMS material actuation in a nonuniform magnetic field and include the reduced-order model for real-time simulations.

## Data Availability Statement

The data that support the findings of this study are openly available in [Magnetic_Cilia_Carpet_Simulations] at [https://github.com/tengz-hang48/Magnetic_Cilia_Carpet_Simulations], reference number [1].

## Supplementary Material

Modeling of single cilia motion

Modeling of metachronal wave motion

Modeling of soft robot rolling

Modeling of soft robot crawling

Jiang_AIS_Supporting_Information
