# Supplementary material for: Numerical Study of Metachronal Wave-Modulated Locomotion in Magnetic Cilia Carpets
Source: Adv Intell Syst. Author manuscript; Available in PMC 2023 Oct 26. (PMC10601495; doi:10.1002/aisy.202300212)
Supplement: Jiang_AIS_Supporting_Information [file NIHMS1929754-supplement-Jiang_AIS_Supporting_Information.pdf]

Supporting Information

Numerical Study of Metachronal Wave Modulated Locomotion in Magnetic Cilia Carpets

*Hao Jiang, Hongri Gu, Bradley J. Nelson, Teng Zhang**

Hao Jiang
Department of Mechanical and Aerospace Engineering, Syracuse University, Syracuse, NY 13244, USA
BioInspired Syracuse, Syracuse University, Syracuse, NY 13244, USA
Email: hjiang37@syr.edu
Hongri Gu University of Konstanz, Konstanz, Germany
Email: hongri.gu@uni-konstanz.de
Bradley J. Nelson
Institute of Robotics and Intelligent Systems, ETH Zurich, Zurich, Switzerland
Email: bnelson@ethz.ch
Teng Zhang
Department of Mechanical and Aerospace Engineering, Syracuse University, Syracuse, NY 13244, USA
BioInspired Syracuse, Syracuse University, Syracuse, NY 13244, USA
Email: tzhang48@syr.edu

1. Model validation

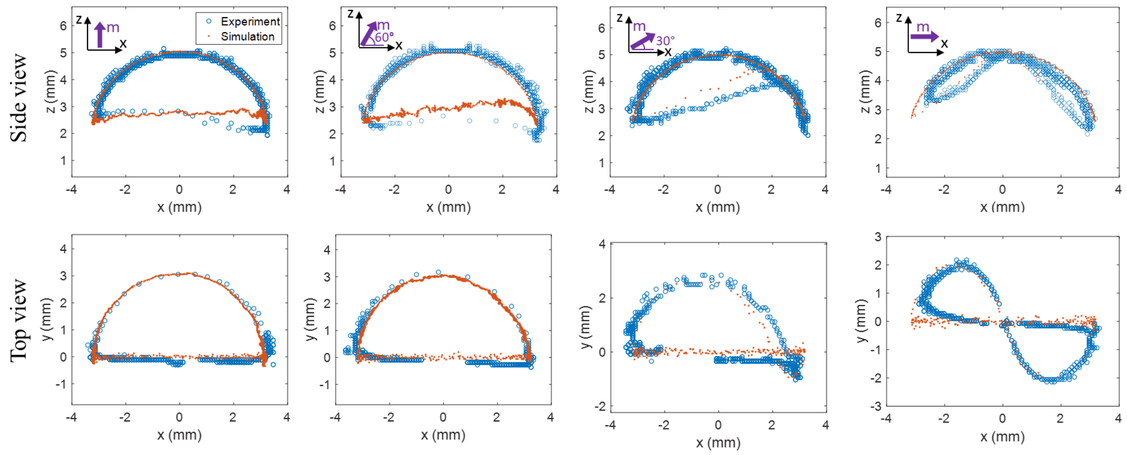

Figure S1. Comparison of simulation and experiential tracking data of the artificial cilia.

2. Supporting Video Description

Video S1 Modeling of single cilia motion.

Video S2 Modeling of metachronal wave motion.

Video S3 Modeling of soft robot rolling.

Video S4 Modeling of soft robot crawling.
